# New Users of Antipsychotics Among Children and Adolescents in 2008–2017: A Nationwide Register Study

**DOI:** 10.3389/fpsyt.2020.00316

**Published:** 2020-04-24

**Authors:** Eveliina Varimo, Leena K. Saastamoinen, Hanna Rättö, Hannu Mogk, Eeva T. Aronen

**Affiliations:** ^1^ Child Psychiatry, University of Helsinki and Helsinki University Hospital, Helsinki, Finland; ^2^ Laboratory of Developmental Psychopathology, Pediatric Research Center, Children's Hospital, University of Helsinki and Helsinki University Hospital, Helsinki, Finland; ^3^ Research Unit, The Social Insurance Institution, Helsinki, Finland; ^4^ Department of Adolescent Psychiatry, University of Helsinki and Helsinki University Hospital, Helsinki, Finland

**Keywords:** antipsychotics, quetiapine, incidence, children, adolescents

## Abstract

**Introduction:**

Recently, prescribing antipsychotics for children and adolescents has been increasing in many countries. These drugs are often prescribed off-label, although antipsychotics have been associated with adverse effects. We determined the recent incidence of antipsychotic use among children and adolescents in Finland.

**Methods:**

Finnish National Prescription Register including all Finnish inhabitants receiving reimbursement for pharmaceuticals was searched for subjects of 1 to 17 years of age who had started an antipsychotic drug between January 1, 2008, and December 31, 2017 (n = 26,353). Between 2008 and 2017, the range of number of Finnish children and adolescents aged 1 to 17 years was 1.01 to 1.03 million/year. The incidence was calculated by dividing the number of new users by all age- and sex-matched Finnish inhabitants in the year.

**Results:**

Between 2008 and 2017, the incidence of antipsychotic use among children and adolescents increased from 2.1 to 3.8 per 1000 individuals, respectively. In children aged 7 to 12 years, the incidence of antipsychotic use 1.4-folded (from 1.9 (95% CI: 1.8–2.0) to 2.7 (95% CI: 2.5–2.9) per 1000) with a cumulative increase of 0.2% per year (χ^2^ = 51.0, *p* < 0.0001). In adolescents aged 13 to 17 years, the incidence 2.2-folded (from 4.3 (95% CI: 4.1–4.5) to 9.4 (95% CI: 9.1–9.8) per 1000) with a cumulative increase of 0.6% per year (χ^2^ = 590.3, *p* < 0.0001). The increase in the incidence of use was steeper in girls (2.3-fold) than in boys (1.4-fold) (χ^2^ = 85.6, *p* < 0.0001), especially between 2015 and 2017 (1.6-fold and 1.2-fold, respectively) (χ^2^ = 151.7, *p* < 0.0001). The year 2011 was the turning point when the incidence in girls exceeded the incidence in boys, and the incidence of quetiapine use exceeded that of risperidone use.

**Conclusions:**

The incidence of antipsychotic use increased between 2008 and 2017, especially in adolescent girls. The use of quetiapine increased, although it has few official indications in children and adolescents. Future studies should investigate the reasons for increasing use of antipsychotics, especially quetiapine, in children and adolescents.

## Introduction

The prescribing of psychotropic drugs in children and adolescents has increased in many countries during the last decade (1–4). This change is mainly due to the growing use of stimulants (2), but the use of antipsychotic drugs is also on the rise (2, 3, 5–7). The prevalence of antipsychotic use in children and adolescents varies from 0.5 to 30.8 per 1000 individuals in different countries (6). Between 2005 and 2012, the prevalence of antipsychotic drug use in children and adolescents increased globally, whereas at the same time in the US the prevalence slightly declined (8). It is not clear, however, if the increase in the prevalence of antipsychotic use is a result of increase of new antipsychotic treatments.

Second-generation antipsychotics (SGAs) have been associated with fewer serious adverse effects, such as extrapyramidal symptoms and tardive dyskinesia, than first-generation antipsychotics (FGAs) (9). Thus, clinicians seem to have assumed that SGAs are a safer treatment regime than FGAs in children and adolescents (10), contributing to the increase in use of SGAs in many countries (6). The prescribing of FGAs in patients aged under 18 years in the US has simultaneously steadily diminished, whereas in Europe FGAs remain popular (9). SGAs have multiple side effects (11, 12). Recent studies have shown that SGAs may be associated with weight gain, increased appetite, obesity, type 2 diabetes, cardiovascular morbidity, abnormal involuntary movements, and sedation (7, 9, 11, 13–16).

In children and adolescents, antipsychotic drugs are frequently used off-label (17, 18). In subjects under 18 years of age, 69% to 92% of the antipsychotics are prescribed without official indications (3, 19, 20). In the US and Europe, the three most commonly prescribed SGAs are in descending order risperidone, quetiapine, and aripiprazole (6, 16, 21). Risperidone has only three official indications in children and adolescents in the US and in the EU (schizophrenia in 14- to 17-year-olds, bipolar I disorder with manic or mixed episodes in 10- to 17-year-olds, and treatment of aggression and irritability in autistic disorder in 5- to 17-year-olds) (22). Conversely, the only official indication in children and adolescents for risperidone in Finland is a maximum of six weeks' use for aggressive behavior in intellectually disabled children aged over 5 years, whereas quetiapine has no official indication in drug treatment of Finnish children and adolescents. Aripiprazole is indicated in Finnish children and adolescents to treat schizophrenia (age above 15 years) or bipolar I disorder for less than 12 weeks (age above 13 years). The fore-mentioned indications are obtained from the summaries of product characteristics accepted by medical authorities (Finnish Medicines Agency, European Medicines Agency). A retrospective study in US showed that attention-deficit hyperactivity disorder (ADHD) was the most common diagnosis in children and adolescents treated with SGAs (17). Other frequent diagnoses in SGA users were conduct disorders, autism, mood disorders, bipolar disorder, tic, Tourette syndrome, aggression, insomnia, and anxiety disorders (1, 3, 16, 23, 24). These data suggest that antipsychotics may be prescribed liberally for many conditions. It is not clear whether the increase in the use of SGAs is a result of an increased number of new antipsychotic users. Remarkably, few studies have investigated the incidence of antipsychotic use in children and adolescents (7, 25–27), and it can only be comprehensively studied in countries with nationwide registers on drug prescriptions (2, 7).

In this nationwide Finnish register study, we report changes in the incidence of antipsychotic use in children and adolescents between 2008 and 2017. Little is known on the incidence of antipsychotic use in the recent years, and the changes in the number of new users of antipsychotic is reflected more precisely by incidence rather than prevalence. Specifically, we evaluate how starting a new antipsychotic drug has changed in different age groups and between sexes. In new antipsychotic users, we characterize frequency of used substances, and diagnoses in children and adolescents with severe mental and behavioral disorders.

## Materials and Methods

All Finnish residents (approximately 5.5 million) are covered under the National Health Insurance, and drug reimbursements are recorded in the Finnish National Prescription Register maintained by the Social Insurance Institution of Finland (SII). The register contains information on the patient and the prescription. We collected data on patients` birth date, gender, dispensing date of the prescription, place of residence, and reimbursement. In the register, all medications are coded according to the Anatomical Therapeutic Chemical (ATC) classification system. We extracted data on the subjects who received reimbursements for antipsychotic medication (all ATC N05A codes). Although, lithium is not an antipsychotic, it was included in the analyses since it is included in the group of antipsychotics in the ATC classification (N05AN01) and it's uses are similar to that of antipsychotics', although more narrow. The definition of on- and off-label SGA use was based on the indications in the summaries of product characteristics accepted by medical authorities (Finnish Medicines Agency, European Medicines Agency). The age of the patient plays a significant role for indications in the summaries of product characteristic. Most of the antipsychotics are not indicated for patients under 18 years.

Between 2008 and 2017, the yearly mean of all 1 to 17 year old Finnish inhabitants was 1.02 million (range, 1.01–1.03 million/year). The study population included children and adolescents aged between 1 and 17 years, who had received antipsychotic drug reimbursements between January 1, 2008, and December 31, 2017, in Finland (n = 70,012). We included all reimbursed antipsychotic prescriptions including small packages in the analyses. We defined a new user (*i.e.* incidence) as a subject who had not filled a prescription of antipsychotics in the previous 730 days. Thus, to cover the wash-out period for new users in 2008, we searched the data from 1 January 2006 to 31 December 2007. The date of the initial purchase was determined as the index date. Subjects' age at treatment initiation was calculated as the difference between birth date and index date. The incidence was calculated by dividing the number of new users by all age- and sex-matched Finnish inhabitants in the year.

To describe the new antipsychotic users further, we gathered register-based diagnostic information on the children and adolescents with severe mental and behavioral disorders from the Register of Disability Benefits of SII. In Finland, children and adolescents with a severe somatic or a psychiatric disorder (mental and behavioral disorders) causing disability and who need regular care, attention, and rehabilitation are entitled to a disability allowance. In 2017, according to SII statistic database Kelasto 3.7% of Finnish children and youth under 16 years of age received disability allowance (www.kela.fi/web/en/statistical-database-kelasto). Of these children 56% had a psychiatric disorder diagnosis. During the last 10 years, the number of the yearly recipients has remained constant. The allowance is intended to provide monetary support for the families of children with disabilities. In the register, the diagnoses of cases with the allowance are coded according to the International Classification of Disease, 10th Revision (ICD-10) and are categorized into primary and secondary diagnoses, the former of which was used in the analyses.

**Figure 1:**
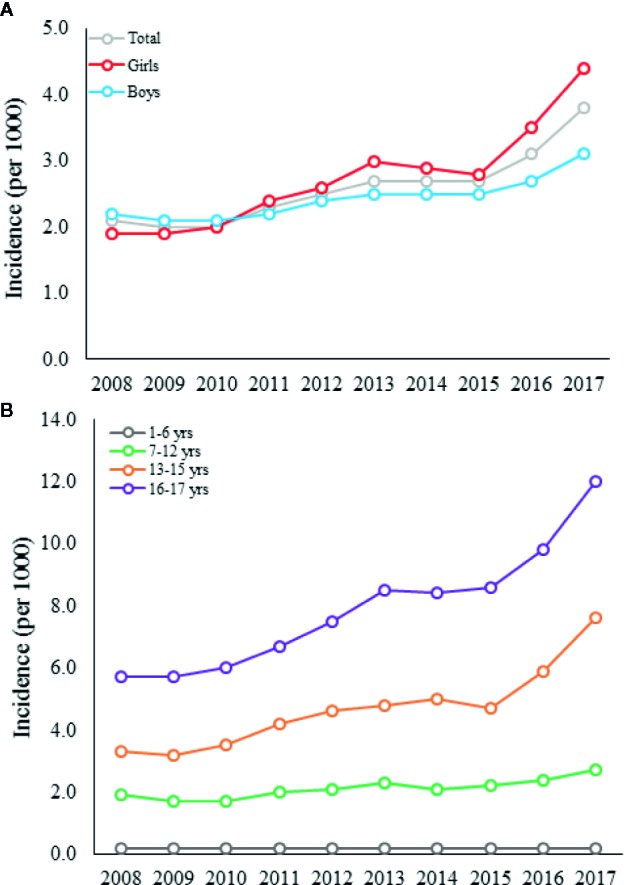
The incidence of antipsychotic use among all children and adolescents **(A)** and in different age groups **(B)** in Finland between 2008 and 2017.

The study population was divided into four age groups (1–6, 7–12, 13–15, and 16–17 years) (**Figure 1B**). The season of treatment initiation was categorized into summer (June-August), autumn (September-November), winter (December-February), and spring (March-May). The drug reimbursement rate was divided into two categories: those with a reimbursement rate of 40% (Basic Refund Category) and to those with a reimbursement rate of 100% (Special Refund Category). The latter group included intellectually disabled persons with aggressive behavior, children and adolescents diagnosed with psychoses or depressive disorders with psychosis or mania.

The study was based only on register data, and thus, no permission from an Ethics Board was required according to the Finnish Medical Research Act. The Social Insurance Institution of Finland approved the use of data for the study.

### Statistical Analyses

The incidence of antipsychotic drug use with 95% confidence interval (CI) was calculated by dividing the number of new users by all age- and sex-matched Finnish inhabitants in the year (data obtained from SII). Additionally, we determined a cumulative growth (% per year) in new antipsychotic users. The cumulative growth was calculated by dividing the number of new users by the number of subjects at risk during the follow-up period. Comparisons between years, sexes, and age groups were performed with Chi-Square test or Fisher's exact test. Statistical analyses were performed with SPSS statistical software for Windows (version 22.2.2, Chicago, IL, USA). Statistical significance was set at P < 0.05.

## Results

Between 2008 and 2017, antipsychotic drug was started in 26 353 subjects (*i.e*. new users), 12 715 (48.2%) of whom were boys. Among the new users, the largest age group was adolescents aged 16 to 17 years (n = 9,813, 37.2%), followed by adolescents aged 13 to 15 years (n = 8,429, 32.0%) and children aged 7 to 12 years (n = 7,470, 28.3%). For additional background data, the prevalences of antipsychotic use in children and adolescents between 2008 and 2017 are presented in **Supplementary Figure 1**.

### Change in Incidence of Antipsychotic Use

Between 2008 and 2017, the incidence of antipsychotic use among children and adolescents increased from 2.1 to 3.8 per 1000 individuals, respectively (**Figure 1A**). The incidence of antipsychotic use 1.4-folded (from 1.9 (95% CI: 1.8–2.0) to 2.7 (95% CI: 2.5–2.9) per 1000) with a cumulative increase of 0.2% per year in children aged 7 to 12 years (χ^2^ = 51.0, *p* < 0.0001). In adolescents aged 13 to 17 years, the incidence 2.2-folded (from 4.3 (95% CI: 4.1–4.5) to 9.4 (95% CI: 9.1–9.8) per 1,000) with a cumulative increase of 0.6% per year (χ^2^ = 590.3, *p* < 0.0001) (**Figure 1B**).

Only 641 (2.4%) of the new users were under 7 years old, and 500 (78.0%) of these were boys. In this subgroup, the change in the incidence of antipsychotic use did not increase significantly between 2008 and 2017 (from 0.2 (95% CI: 0.2–0.3) to 0.2 (95% CI: 0.2–0.3) per 1,000, the cumulative increase was 0.02% per year) (χ^2^ = 1.0, p = 0.31) (**Figure 1B**). In all age groups, the increase in incidence between 2008 and 2017 was stronger in girls than in boys, as the incidence of antipsychotic use 2.3-folded in girls and 1.4-folded in boys (χ^2^ = 85.6, *p* < 0.0001) (**Table 1**). In detail, the largest increase in the incidence was observed between 2015 and 2017, and during this period, the incidence 1.6-folded in girls and 1.2-folded in boys (χ^2^ = 151.7, *p* < 0.0001) with a cumulative increase of 0.4% and 0.3% per year, respectively (**Figure 1A**). Between 2013 and 2015, the incidence remained constant in both sexes (**Figure 1A**). Thereafter, the incidence increased substantially, especially in girls (**Figure 1A**). The year 2011 was the turning point when the incidence in girls exceeded that in boys, and the incidence of quetiapine use exceeded that of risperidone use.

**Table 1:** The incidence of antipsychotics and starting season among children and adolescents in Finland between 2008 and 2017.

	2008		2009		2010		2011		2012		2013		2014		2015		2016		2017	
	**Number**	**Incidence (95%CI)**	**Number**	**Incidence (95% CI)**	**Number**	**Incidence (95% CI)**	**Number**	**Incidence (95% CI)**	**Number**	**Incidence (95% CI)**	**Number**	**Incidence (95% CI)**	**Number**	**Incidence (95% CI)**	**Number**	**Incidence (95% CI)**	**Number**	**Incidence (95% CI)**	**Number**	**Incidence (95% CI)**
Drug																				
Quetiapine	809	0.8 (0.7–0.9)	821	0.8 (0.7–0.9)	966	0.9 (0.8–1.0)	1182	1.2 (1.1–1.3)	1308	1.3 (1.2–1.4)	1445	1.4 (1.3–1.5)	1442	1.4 (1.3–1.5)	1369	1.3 (1.2–1.4)	1721	1.7 (1.6–1.8)	2275	2.2 (2.1–2.3)
Risperidone	1083	1.0 (0.9–1.1)	1008	1.0 (0.9–1.1)	915	0.9 (0.8–1.0)	986	1.0 (0.9–1.1)	1044	1.0 (0.9–1.1)	1128	1.1 (1.0–1.2)	1060	1.0 (0.9–1.1)	1083	1.1 (1.0–1.2)	1164	1.1 (1.0–1.2)	1219	1.2 (1.1–1.3)
Olanzapine	86	0.08 (0.08–0.08)	73	0.07 (0.07–0.07)	51	0.05 (0.05–0.05)	56	0.05 (0.05–0.05)	56	0.05 (0.05–0.05)	48	0.05 (0.05–0.05)	77	0.08 (0.08–0.08)	100	0.1 (0.1–0.1)	97	0.1 (0.1–0.1)	148	0.2 (0.2–0.2)
Aripiprazole	11	0.01 (0.01–0.01)	30	0.03 (0.03–0.03)	29	0.03 (0.03–0.03)	41	0.04 (0.04–0.04)	39	0.04 (0.04–0.04)	33	0.03 (0.03–0.03)	48	0.05 (0.05–0.05)	64	0.06 (0.06–0.06)	92	0.09 (0.09–0.09)	113	0.1 (0.1–0.1)
Haloperidol	35	0.03 (0.03–0.03)	36	0.04 (0.04–0.04)	39	0.04 (0.04–0.04)	42	0.04 (0.04–0.04)	42	0.04 (0.04–0.04)	29	0.03 (0.03–0.03)	24	0.02 (0.02–0.02)	33	0.03 (0.03–0.03)	37	0.04 (0.04–0.04)	18	0.02 (0.02–0.02)
Chlorprothixene	37	0.04 (0.04–0.04)	30	0.03 (0.03–0.03)	29	0.03 (0.03–0.03)	24	0.02 (0.02–0.02)	29	0.03 (0.03–0.03)	18	0.02 (0.02–0.02)	10	0.01 (0.01–0.01)	11	0.01 (0.01–0.01)	9	0.009 (0.009–0.009)	13	0.01 (0.01–0.01)
Levomepromazine	28	0.03 (0.03–0.03)	9	0.009 (0.009–0.009)	23	0.02 (0.02–0.02)	21	0.02 (0.02–0.02)	13	0.01 (0.01–0.01)	19	0.02 (0.02–0.02)	15	0.01 (0.01–0.01)	25	0.02 (0.02–0.02)	13	0.01 (0.01–0.01)	24	0.02 (0.02–0.02)
Clozapine	10	0.01 (0.01–0.01)	4	0.004 (0.004–0.004)	8	0.008 (0.008–0.008)	7	0.007 (0.007–0.007)	7	0.007 (0.007–0.007)	11	0.01 (0.01–0.01)	16	0.02 (0.02–0.02)	6	0.006 (0.006–0.006)	6	0.006 (0.006–0.006)	7	0.007 (0.007–0.007)
Pimozide	10	0.01 (0.01–0.01)	13	0.01 (0.01–.0.01)	10	0.01 (0.01–.0.01)	7	0.007 (0.007–0.007)	3	0.003 (0.003–0.003)	12	0.01 (0.01–.0.01)	8	0.008 (0.008–0.008)	0	0	3	0.003 (0.003–0.003)	0	0
Perphenazine	6	0.006 (0.006–0.006)	3	0.003 (0.003–0.003)	7	0.007 (0.007–0.007)	5	0.005 (0.005–0.005)	5	0.005 (0.005–0.005)	8	0.008 (0.008–0.008)	4	0.004 (0.004–0.004)	11	0.01 (0.01–0.01)	4	0.004 (0.004–0.004)	2	0.002 (0.002–0.002)
Lithium	3	0.003 (0.003–0.003)	3	0.003 (0.003–0.003)	4	0.004 (0.004–0.004)	2	0.002 (0.002–0.002)	3	0.003 (0.003–0.003)	4	0.004 (0.004–0.004)	3	0.003 (0.003–0.003)	2	0.002 (0.002–0.002)	3	0.003 (0.003–0.003)	4	0.004 (0.004–0.004)
Other	23		6		11		9		5		2		4		0		4		0	
**Starting season**																				
June-August	454	0.4 (0.4–0.4)	392	0.4 (0.4–0.4)	447	0.4 (0.4–0.4)	472	0.5 (0.5–0.5)	493	0.5 (0.5–0.5)	472	0.5 (0.5–0.5)	495	0.5 (0.5–0.5)	490	0.5 (0.5–0.5)	572	0.6 (0.6–0.6)	654	0.6 (0.6–0.6)
September-November	562	0.5 (0.5–0.5)	512	0.5 (0.5–0.5)	547	0.5 (0.5–0.5)	689	0.7 (0.7–0.8)	733	0.7 (0.7–0.8)	815	0.8 (0.7–0.9)	701	0.7 (0.7–0.8)	748	0.7 (0.7–0.8)	996	1.0 (0.9–1.1)	1093	1.1 (1.0–1.2)
December-February	599	0.6 (0.6–0.6)	554	0.5 (0.5–0.5)	542	0.5 (0.5–0.5)	646	0.6 (0.6–0.6)	663	0.7 (0.7–0.8)	737	0.7 (0.7–0.8)	763	0.7 (0.7–0.8)	746	0.7 (0.7–0.8)	841	0.8 (0.8–0.9)	1062	1.0 (0.9–1.1)
March-May	526	0.5 (0.5–0.5)	578	0.6 (0.6–0.6)	556	0.5 (0.5–0.5)	575	0.6 (0.6–0.6)	665	0.7 (0.7–0.8)	733	0.7 (0.7–0.8)	752	0.7 (0.7–0.8)	720	0.7 (0.7–0.8)	744	0.7 (0.7–0.8)	1014	1.0 (0.9–1.1)

### The Diagnoses of Children and Adolescents With Disability Allowance in New Antipsychotic Users

Between 2008 and 2017, altogether 8,464 (32.1%) of the new antipsychotic users had received disability allowance. During the study period, the three most common mental and behavioral disorder (F00-F99) diagnoses related to being granted a disability allowance were hyperkinetic disorders (F90) (n = 1,532, 20.4%), pervasive developmental disorders (F84) (n = 1,147, 15.3%), and mixed disorders of conduct and emotions (F92) (n = 654, 8.7%) (**Supplementary Table 1**). These diagnoses accounted for 44.4% of all mental and behavioral disorder diagnoses in children and adolescents with disability allowance. Of all mental and behavioral disorder diagnoses in children and adolescents with disability allowance, 5,252 (70.1%) were diagnoses without official indications for antipsychotic treatment. In new antipsychotic users aged under 7 years, 467 (74.1%) were granted a disability allowance, and the three most common diagnoses were pervasive developmental disorders (F84), mixed specific developmental disorders (F83), and hyperkinetic disorders (F90) (**Supplementary Table 1**). Between 2008 and 2017, we observed a significant increase in hyperkinetic disorders (F90) (χ^2^ = 6.4, *p* = 0.01) and pervasive developmental disorders (F84) (χ^2^ = 7.2, *p* = 0.01) in new antipsychotic users aged 7 to 12 years with disability allowance (**Supplementary Table 2**). At the same time, the frequency of pervasive developmental disorders (F84) (χ^2^ = 4.9, *p* = 0.03) and depressive episode (F32) (χ^2^ = 8.8, *p* = 0.003) increased in new antipsychotic users aged 13 to 17 years (**Supplementary Table 2**).

**Figure 2:**
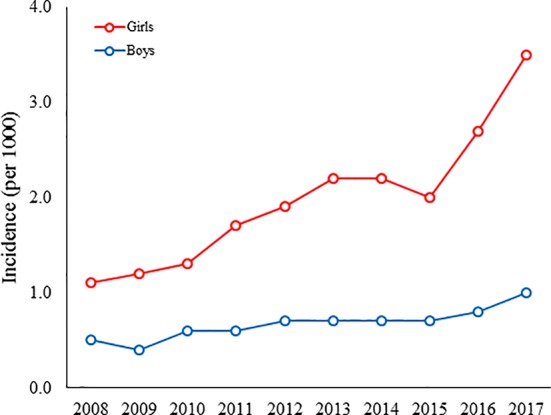
The incidence of quetiapine use among Finnish boys and girls aged 1 to 17 years between 2008 and 2017.

Between 2008 and 2017, the four most commonly first purchased antipsychotic drugs in children and adolescents who started antipsychotics *(i.e. new users*) were quetiapine (n = 13 338, 50.6% of all antipsychotic users), risperidone (n = 10 690, 40.6%), olanzapine (n = 792, 3.0%), and aripiprazole (n = 500, 1.9%) (**Table 1**). These four substances accounted for the majority of new antipsychotic prescriptions (n = 25 320; 96.1%). During the study period, quetiapine was initiated more frequently in girls than in boys, and the increase of incidence was steepest during the last years (**Figure 2**). Before the year 2011, risperidone was the most commonly prescribed new antipsychotic; thereafter, the incidence of quetiapine prescriptions has exceeded risperidone. During the study period risperidone was the most prescribed new antipsychotic drug in subjects aged under 7 years (n = 583, 91.0%) (**Supplementary Table 1**). New antipsychotic drug was initiated most commonly in autumn (September-November) (n = 7396, 28.1%, χ^2^ = 761.0, *p* < 0.0001). During the study period, 333 (1.3%) prescriptions were reimbursed in Special Refund category and the rest in Basic Refund category. Of the 333 prescriptions, 288 (86%) were prescribed to persons with psychoses or depressive disorders with psychosis or mania and 45 (24%) for aggressive behavior in intellectually disabled children.

## Discussion

In this nationwide register study, we found that the incidence of antipsychotic use has increased in children and adolescents during the last 10 years, with the increase being particularly strong between 2015 and 2017. From 2011, the incidence in girls exceeded that in boys, and at the same time the incidence of quetiapine treatment exceeded that of risperidone treatment.

The incidence of antipsychotic use increased in children and adolescents in Finland between 2008 and 2017. A similar rise in the incidence of antipsychotic use aged under 18 years has been reported in Dutch population (7). In our study, the largest incidence of antipsychotic use was observed in adolescents aged 16 to 17 years, followed by subjects aged 13 to 15 years and 7 to 12 years. The increase in incidence of antipsychotic use may have several reasons. Nowadays, psychiatric outpatient care is more easily accessible for children and adolescents, and the views of parents and physicians regarding the prescribing of antipsychotic drugs have been reported to be more favourable (7, 19, 25, 28). Importantly, antipsychotic drugs have been used more often off-label in subjects with hyperkinetic and conduct disorders (3, 17, 25, 29). We speculate clinicians may also prescribe antipsychotics, especially quetiapine, in children and adolescents to treat depression and anxiety disorders and insomnia reflecting the use of quetiapine in adult population (30). Finally, the limited availability of psychotherapy and the greater commitment that it entails relative to prescribing medication could favor the use of antipsychotic drugs (17, 28).

The year 2011 was the turning point when the incidence of antipsychotic use in girls exceeded that in boys, and this trend remained until the end of the follow-up. In Finland, the prevalence of emotional problems has increased more in adolescent girls than in adolescent boys during the last 20 years (31). Conversely, the use of antipsychotics in all age groups has been reported to be more common in boys than in girls (7, 17, 32), although the gap between the sexes narrows with age in adolescence (17, 32). Similar to our results, a recent Dutch register study reported that the incidence on antipsychotic use in girls exceeded that in boys in 2015 (7). Our finding of the rapid increase in the incidence of antipsychotic use in girls may be a result of multiple short treatment periods or antipsychotics being prescribed to girls to treat symptoms or mental disorders such as insomnia, anxiety disorders, mood disorders, and agitation (3, 19, 20, 24, 33). Additionally, previous studies have shown that in all age groups boys are treated with antipsychotics for longer periods than girls (7). Future studies should investigate the duration of the antipsychotic treatment and how the medication follow-up is actualized. It is important to pay careful attention to monitoring the harms and benefits of antipsychotic treatment and to discontinue ineffective treatment in children and adolescents (10, 34).

In our study, quetiapine replaced risperidone as the most prescribed new antipsychotic drug after 2011. A similar turning point (2012) was observed in Denmark (35). Our finding is consistent with a previous study that reported quetiapine as the most common antipsychotic drug in adolescents older than 14 years and found a significant increase in the frequency of quetiapine use in subjects under 14 years of age (32). In our study, quetiapine was initiated more frequently in girls than in boys, especially in girls during the last two years of the study period. Based on our clinical expertise we hypothesise that low dose (*i.e.,* 25–75 mg daily) quetiapine was prescribed in short treatment durations in girls to treat anxiety disorders and insomnia. To this end, a previous study reported a high use of quetiapine for insomnia in adolescent girls admitted to psychiatric inpatient care (36). Furthermore, the study found that quetiapine was prescribed for only insomnia in 57% of the admitted girls, and 25% were diagnosed with anxiety disorder, depressive disorder, eating disorder, or personality disorder (36). This is remarkable as quetiapine has only a few official indications in children and adolescents (schizophrenia 13 years and older, bipolar disorder manic and mixed episodes 10 years and older) (US Food and Drug Administration) in the US. This probably reflects the use of quetiapine in adults, as a Norwegian register study reported that the median daily dose of quetiapine was less than 100 mg, and that only approximately 4% of users received doses and reimbursement consistent with the use of quetiapine for an approved indication (37). Importantly, the use of quetiapine in children and adolescents is associated with a considerable risk of side effects such as weigh gain, increased triglycerides, blood pressure and heart rate, and daytime sedation which may occur early after treatment initiation (15, 38–41). In Finland, quetiapine has no official indications in subjects aged under 18 years, which suggests substantial off-label use of quetiapine in Finnish adolescents.

In children aged 7 to 12 years with disability allowance, hyperkinetic disorders and pervasive developmental disorders were the most common diagnoses in new antipsychotic users, whereas pervasive developmental and depressive disorders were enriched in new users aged 13 to 17 years. In general, the children and adolescents with a more severe disorder need more care and attention and are more likely to be entitled to a disability allowance than the subjects with a less severe disorder. This translates into the children and adolescents in the disability register having on average a more severe diagnostic status than those not in the register. At the same time, we agree that the diagnoses of this subsample may not be generalized to all new SGA users. In the EU and the US, the most common diagnoses in children and adolescents who receive antipsychotics are hyperkinetic disorder, conduct disorder, autism spectrum disorder, mental retardation, depression, and bipolar disorder (7, 17, 25, 28, 32). Conversely, studies in the US and in the Netherlands have reported the two most common diagnoses in new antipsychotic users to be bipolar disorder and schizophrenia (7, 25). Supporting our finding, the incidence of behavioral disorders and autism spectrum disorders have been found to be enriched in early and middle childhood, whereas depression and anxiety disorders increase in adolescence (17, 24, 26). While serious mental disorders, such as schizophrenia and psychotic disorders, have not increased among new antipsychotic users aged under 18 years (25), we and others have observed an increase in prescribing antipsychotic drugs (3, 25).

The main strength of this study is that it is a comprehensive nationwide register study that covers all reimbursed outpatient prescription drug purchases in children and adolescents aged 1 to 17 years. This study focused on incidence rather than prevalence, since the former describes more precisely the changes in the number of new users. The data included a two-year wash-out time to select subjects who were truly new antipsychotic users. The present study includes limitations that should be addressed. The data included only outpatient use of antipsychotics, and non-prescribed medicines were not included indicating that a small number of prescriptions were not recorded in the register. Our data on diagnoses was incomplete, since the data did not include diagnoses for all users, only for users with disability allowance (32.1% of our SGA users). Further, the data did not include the indications for SGA prescriptions. The use of antipsychotics could not be confirmed, and we can only assume that the purchased antipsychotics were used as prescribed. To get more specific information on SGA use, future studies might benefit from combining the data of prescription register and medical records. This study did not address treatment duration of SGA use, which may have changed in children and adolescents during the study period and may partly explain the increase in the incidence of antipsychotic use. This warrants further investigation.

In conclusion, the incidence of antipsychotic use increased during the study period, especially in adolescent girls between 2015 and 2017. In 2011, quetiapine overtook risperidone as the most prescribed antipsychotic, and the incidence of antipsychotic use in girls exceeded that in boys. We observed that antipsychotic drugs were prescribed almost exclusively for off-label indications in children and adolescents. Future studies should investigate the duration of antipsychotic treatments and the reasons for the increased use of antipsychotics, especially quetiapine.

## Data Availability Statement

The data that support the findings of this study are available from the Social Insurance Institution of Finland (SII) but restrictions apply to the availability of these data, which were used under license for the current study, and thus are not publicly available. The data are however available from the authors upon reasonable request and with permission of the SII.

## Author Contributions

EV conducted the analyses and drafted the initial manuscript. LS and EA designed the study, coordinated, and supervised the data collection, and critically reviewed the manuscript. HR and HM contributed to planning the data analyses, reviewed, and revised the manuscript. All authors approved the final manuscript as submitted and agree to be accountable for all aspects of the work.

## Funding

This study was funded by The Social Insurance Institution of Finland (EV personal grant) and Helsinki University Hospital research funds (EV personal grant and EA personal grant (TYH2016202, TYH2017205).

## Conflict of Interest

The authors declare that the research was conducted in the absence of any commercial or financial relationships that could be construed as a potential conflict of interest.
