# Supplementary figures and images for: New Users of Antipsychotics Among Children and Adolescents in 2008–2017: A Nationwide Register Study
Source: Front Psychiatry. 2020 Apr 24;11:316. doi: 10.3389/fpsyt.2020.00316 (PMC7193104; doi:10.3389/fpsyt.2020.00316)

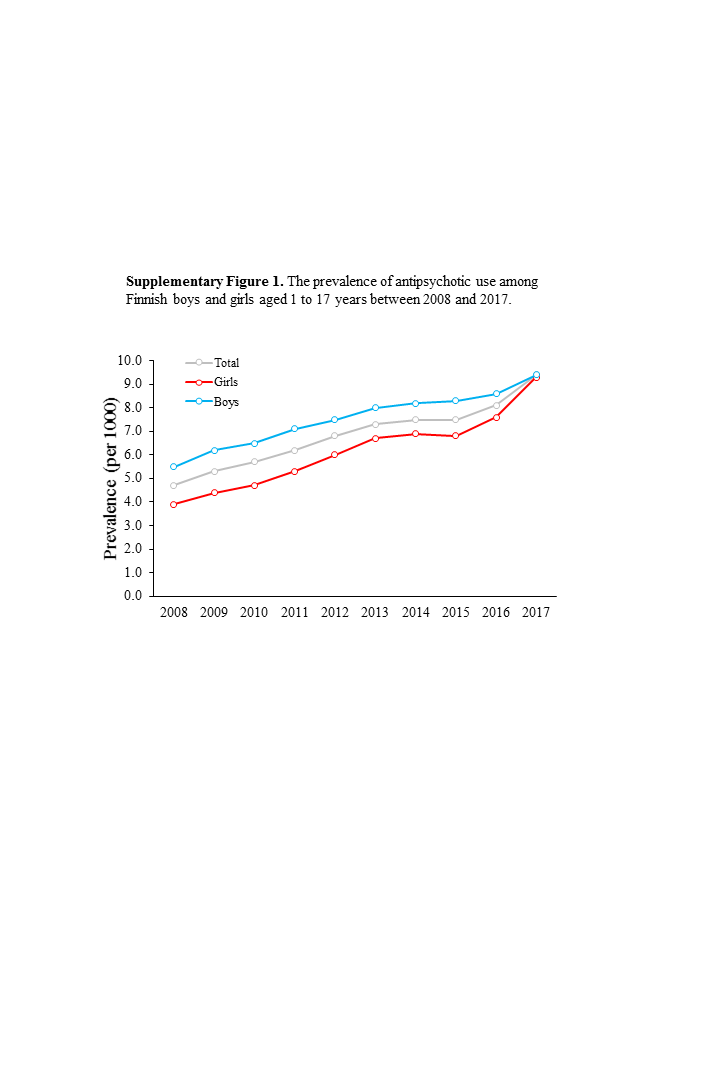

Supplement: Supplementary file 3 [file Image_1.tif]
